# Structural studies of codeinone reductase reveal novel insights into aldo-keto reductase function in benzylisoquinoline alkaloid biosynthesis

**DOI:** 10.1016/j.jbc.2021.101211

**Published:** 2021-09-20

**Authors:** Samuel C. Carr, Megan A. Torres, Jeremy S. Morris, Peter J. Facchini, Kenneth K.S. Ng

**Affiliations:** 1 Department of Biological Sciences, University of Calgary, Calgary, Alberta, Canada; 2 Department of Chemistry and Biochemistry, University of Windsor, Windsor, Ontario, Canada

**Keywords:** X-ray crystallography, biosynthesis, secondary metabolism, enzyme structure, plant biochemistry, site-directed mutagenesis, natural product biosynthesis, structure-function, AKR, aldo-keto reductase, BIA, benzylisoquinoline alkaloid, CHR, chalcone reductase, COR, codeinone reductase, DRR, 1,2 dehydroreticuline reductase, DRS, 1,2 dehydroreticuline synthase, MecgoR, methylecgonone reductase, NISO, neopinone isomerase, REPI, reticuline epimerase, T6ODM, thebaine 6-*O*-demethylase, THS, thebaine synthase

## Abstract

Benzylisoquinoline alkaloids (BIAs) are a class of specialized metabolites with a diverse range of chemical structures and physiological effects. Codeine and morphine are two closely related BIAs with particularly useful analgesic properties. The aldo-keto reductase (AKR) codeinone reductase (COR) catalyzes the final and penultimate steps in the biosynthesis of codeine and morphine, respectively, in opium poppy (*Papaver somniferum*). However, the structural determinants that mediate substrate recognition and catalysis are not well defined. Here, we describe the crystal structure of apo-COR determined to a resolution of 2.4 Å by molecular replacement using chalcone reductase as a search model. Structural comparisons of COR to closely related plant AKRs and more distantly related homologues reveal a novel conformation in the β1α1 loop adjacent to the BIA-binding pocket. The proximity of this loop to several highly conserved active-site residues and the expected location of the nicotinamide ring of the NADP(H) cofactor suggest a model for BIA recognition that implies roles for several key residues. Using site-directed mutagenesis, we show that substitutions at Met-28 and His-120 of COR lead to changes in AKR activity for the major and minor substrates codeinone and neopinone, respectively. Our findings provide a framework for understanding the molecular basis of substrate recognition in COR and the closely related 1,2-dehydroreticuline reductase responsible for the second half of a stereochemical inversion that initiates the morphine biosynthesis pathway.

Opiates are essential and currently irreplaceable medicines for the management of severe pain associated with severe burns, postoperative recovery, cancer treatment, and palliative care (1). Globally, the licit demand for eight billion defined daily doses per year (459 tons of morphine equivalents) is almost entirely supplied by the agricultural production of opium poppy plants in Turkey, Tasmania, and Eastern Europe (2). While several opiate pharmaceuticals are isolated directly from the plant (*e.g.*, morphine and codeine), others are derived from the structurally related, nonmedicinal alkaloid thebaine to yield a suite of semisynthetic opiates with refined pharmacological properties (*e.g.*, oxycodone, hydrocodone, and buprenorphine (2)). Driven by the large capital investment required to establish pharmaceutical manufacturing capacity, coupled with the challenges of sustaining agricultural productivity in an increasingly unpredictable climate and securing global supply chains in a frequently unstable geopolitical environment, recent attention has focused on the potential biosynthesis of medicinal opiates in engineered microorganisms. Heterologous production systems also provide new opportunities to introduce novel enzyme biocatalysts and to direct the flux of metabolites toward specific products through protein and genome engineering, thus offering the possibility of creating novel compounds generally inaccessible through traditional plant breeding or postextraction chemical modification. Key to the success of this emerging synthetic biology strategy is a deep understanding of the genes, enzymes, and pathways that have evolved in opium poppy over tens of millions of years (3).

In opium poppy, the first committed step in morphine biosynthesis is the stereochemical inversion of (*S*)-reticuline to (*R*)-reticuline by reticuline epimerase (REPI) (4, 5). Next, a carbon–carbon phenol-coupling establishes the promorphinan scaffold in salutaridine (CYP719B1; salutaridine synthase; SalSyn), which further undergoes carbonyl reduction (salutaridine reductase; SalR), *O*-acetylation (salutaridine acetyltransferase; SalAT), and allylic rearrangement (thebaine synthase; THS) to form the pentacyclic morphinan structure characteristic of medicinal opiates (6, 7). From thebaine, the major route to morphine proceeds through an initial *O*-demethylation of the B-ring (thebaine 6-*O*-demethylase; T6ODM) producing neopinone (Fig. 1). Following a double-bond rearrangement (neopinone isomerase; NISO), codeinone is reduced to codeine by codeinone reductase (COR). Ultimately, *O*-demethylation of the A-ring (codeine *O*-demethylase; CODM) yields morphine. In an alternative minor route from thebaine, consecutive B-ring and A-ring *O*-demethylations (*i.e.*, CODM followed by T6ODM) form neomorphinone, which is converted to morphine in two steps in parallel with the major route (NISO, COR).

**Figure 1:**
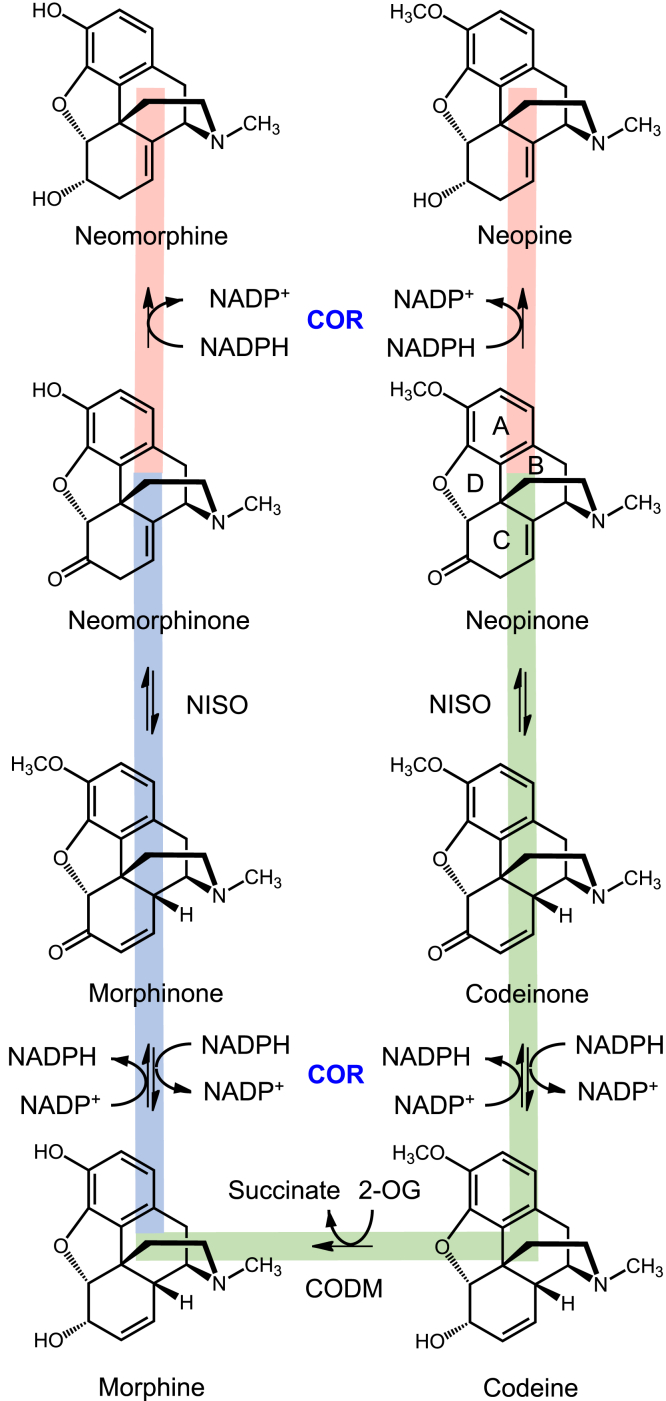
**Morphinan biosynthetic pathway.** Known pathways for the conversion of neomorphinone and neopinone to morphine in *Papaver somniferum*. Both neomorphinone and neopinone are derived from thebaine. In the major pathway (shown in *green*), neopinone is isomerized to codeinone by neopinone isomerase (NISO). Codeinone is reduced to codeine by COR and codeine converted to morphine by codeine *O*-demethylase (CODM). In the minor pathway (shown in *blue*), neomorphinone is isomerized to morphinone by NISO and morphinone reduced by COR to form morphine. COR accepts both neomorphinone and neopinone and converts these substrates irreversibly to neomorphine and neopine, respectively (shown in *red*). Ring assignment used by convention (*i.e.*, A, B, C, D) are indicated on neopinone but apply to all morphinans.

Efficient conversion of neopinone to codeine is critical to opiate biosynthesis and has been identified as a problematic “bottleneck” in achieving high product titers in engineered microorganisms (8). Under physiological conditions *in vitro*, the spontaneous isomerization of codeinone and neopinone establishes an equilibrium whereby the two molecules occur at similar concentrations (9). Although COR is able to reduce the carbonyl moiety in codeinone and neopinone to yield codeine or neopine, respectively, COR can only efficiently catalyze the reverse reaction (*i.e.*, alcohol oxidation) with codeine as the substrate (10). As a result, the inclusion of *COR* in early iterations of opiate-producing engineered microorganisms led to the predominant accumulation of neopine at the expense of codeine or derivatives thereof (8, 10, 11). More recently, the coexpression of *NISO* and *COR* dramatically reduced the undesired formation of neopine to a level near that observed in opium poppy plants (12). NISO accelerates the isomerization of neopinone to codeinone, thus limiting the availability of neopinone for irreversible reduction to neopine by COR. Despite the benefits of including *NISO* to improve the production of desired opiates in engineered microbes, some neopine formation still occurs, which necessitates relatively low levels of *COR* expression and limits the titers of desired opiate products. To overcome this limitation, enzyme engineering efforts targeted a COR mutant with a more desirable activity profile. Unfortunately, a lack of information on the structural elements responsible for substrate recognition in COR has so far limited the success of engineering strategies (12).

COR belongs to the large and well-studied AKR superfamily, which is characterized by the TIM-barrel fold and mechanistically flexible NAD(P)H dependant oxidoreductase activity (13). AKRs are ubiquitous in living organisms and have been divided into 18 subfamilies according to a strict 40% sequence identity cutoff (14). In plants, AKRs contribute to a wide range of processes including detoxification of reactive molecules, iron acquisition, carbon assimilation, and specialized metabolite biosynthesis. The current range of plant metabolites involving AKR enzymes in their biosynthesis includes cardiac glycosides (AKR4C4; AKR4C5), ascorbic acid (15), flavonoids (*e.g.*, AKR4A1, AKR4A3, AKR4A4, AKR4B1), and alkaloids of the monoterpene indole (AKR13D1), tropane (methylecgonone reductase; MecgoR), and benzylisoquinoline (AKR4B3, COR; REPI/DRR) types (reviewed in (16)). Outside of BIA biosynthesis, chalcone reductase (CHR), involved in isoflavonoid biosynthesis (17), and MecgoR (18) are most closely related to COR (54 and 50% amino acid sequence identity, respectively). A structure has been reported only for CHR (19). While several reports describe the use of CHR as a template for the homology modeling of COR, the low sequence identity and ligand-binding site flexibility have limited structure–function insights to general observations concerning an enlarged BIA-binding pocket (19). The COR structure presented here provides novel insights into the substrate specificity of BIA-binding AKR enzymes, which has facilitated the design of COR mutants with a substantially altered substrate preference that favors the production of desired opiates over undesirable by-products.

## Results

### Overall structure

The crystal structure of the COR1.3 isoform (hereafter referred to as COR) was solved using molecular replacement and refined to a resolution of 2.4 Å (Table 1). Even after the conditions used to grow the monoclinic (P2_1_) crystals were extensively optimized, moderately severe anisotropy in the diffraction pattern limited the quality of data that could be measured. As a result, the data collection statistics, especially beyond 2.8 Å resolution, indicate the weakness of the diffraction when the X-ray beam was oriented normal to the thin face of the plate-like crystals. Because the quality of electron density maps was significantly improved when including data to a resolution of 2.4 Å resolution (Fig. S1), the weak and anisotropic data between 2.8 and 2.4 Å resolution were included in the refinement and map calculations even though, as a result, the overall R-factors in both the data collection and refinement statistics appear slightly higher than ideal values. Six copies of COR (three homodimeric complexes, each with *C*_*2*_ point group symmetry) are found in the asymmetric unit and labeled as chains A–F. The following residues did not have sufficient electron density to be modeled with confidence: N-terminal residues 1–4 in chains A-F; Loop A residues 126–133 in chains A, E and F, 126–136 in chain B, 126–132 in chains C and chain D; Loop C residues 302–312 in chain C; C-terminal residues 318–321 in chains A, B, D–F and residues 317–321 in chain C.

**Table 1:** Crystallographic data collection and refinement statistics for *Papaver somniferum* COR1.3

Data collection and refinement statistics	Apo-COR
Data collection statistics	
PDB code	7MBF
Space group	P2_1_
Unit cell dimensions	
⍺, b, c (Å)	78.937, 90.937, 144.801
⍺, β, y (°)	90, 93.529, 90
Wavelength (Å)	0.97946
Resolution (Å)	50.0–2.4 (2.49–2.40)
*R*_*sym*_	0.214 (0.958)
*CC*_*1/2*_	0.966 (0.523)
*I*/σ	2.8 (0.58)
Completeness (%)	86.2 (47.6)
Redundancy	2.9 (1.65)
Refinement	
Resolution (Å)	50.0–2.4
Unique reflections	67,185
*R*_*work*_*/R*_*free*_	0.2194/0.2773
Total no. of atoms	14,524
Protein atoms	14,260
Water atoms	164
Average *B*-factors (protein)	50.4
Average *B*-factors (water)	36.4
r.m.s.d. from ideal geometry	
Bond length	0.002
Bond angles	0.49
Ramachandran outliers (%)	0.11
Ramachandran favored (%)	96.69
MolProbity score	1.56
Clashscore	6.32

**Figure 2:**
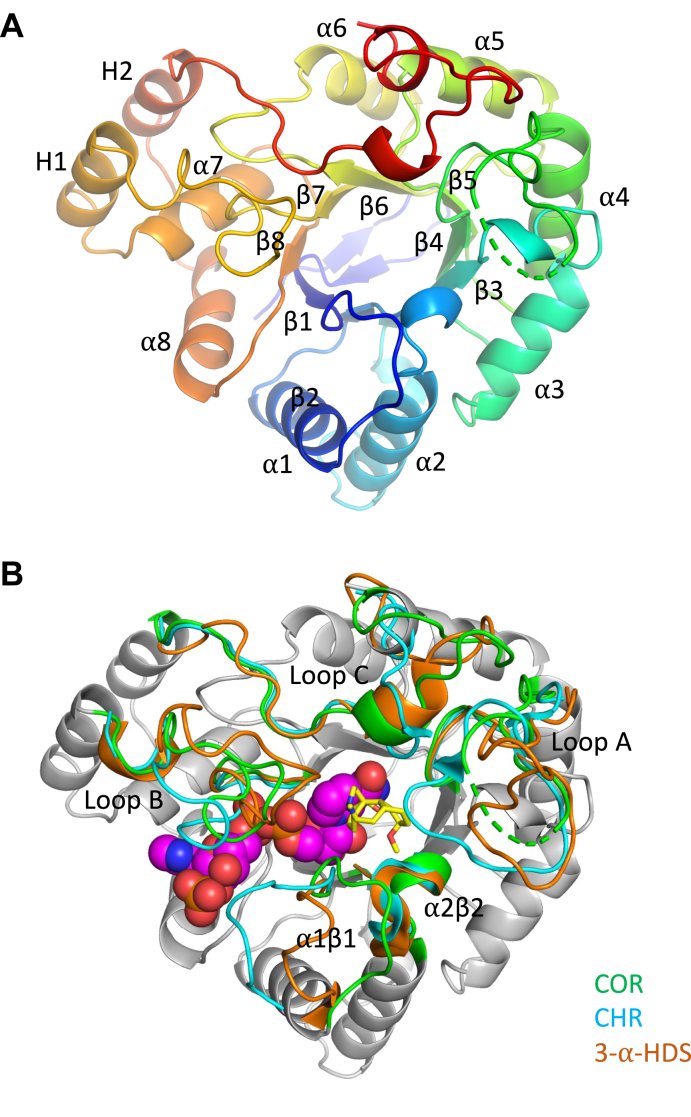
**COR crystal structure.***A*, view looking down from the top of the TIM-barrel of COR. Only the α-carbon backbone is drawn. The rainbow coloring scheme starts at the N-terminus in *blue* to the C-terminus in *red*. Helices and β strands are numbered according to convention. *B*, overlay of loops A, B, C, ⍺1β1, and ⍺2β2 of COR in *green*, CHR in *cyan* (1ZGD), and 3-α-HDS in *orange* (1J96) and the COR main fold in *gray* with NADP^+^ in *magenta* superimposed from CHR-NADP+ complex structure (1ZGD), and codeine in *yellow* superimposed from induced-fit docking. The specific isoform crystallized was COR1.3, which shows greater than 96–99% identity with all other known isoforms (10).

The overall three-dimensional structure of COR reveals a TIM-barrel fold (Figs. 2*A* and S2) common to the AKR superfamily (14), in which eight parallel β-strands form a central barrel surrounded by eight α-helices to form the (α/β)_8_ barrel. The bottom of the barrel is capped with an N-terminal β-hairpin, and the side of the barrel is lined by two auxiliary α-helices (H1 and H2). Three large loops (Fig. 2*B*) play major roles in defining substrate specificity (14, 20) and are named loops A (Ile-118-Tyr-143), B (His-213-Lys-232), and C (Glu-291-Glu-314) by convention. Loops A and C contribute solely to forming the substrate-binding pocket while loop B contributes to both substrate and cofactor binding. Two smaller loops named β1α1 (Gly-23-Glu-33) and β2α2 (Asp-51-Glu-59) also contribute to substrate specificity and catalysis. The β1α1 loop contributes to both the substrate and cofactor binding pockets (Fig. 2*B*), whereas the β2α2 loop forms part of the substrate binding pocket and contributes the two key catalytic residues Asp-51and Tyr-56. These two residues and two additional residues (Lys-86 and His-119) occupy positions typical of the canonical catalytic tetrad seen in all AKRs.

Although 1 mM NADPH and 1 mM codeine were present during crystallization, no electron density corresponding to NADPH was seen. Although weak electron density was seen in the active site at a location expected for codeine, this electron density was too weak to define a precise mode of binding. The crystal structure described in this paper thus represents an apoenzyme state with a well-defined binding pocket for the NADPH cofactor similar to that seen for other AKRs binding to either NADPH or NADH. The putative binding site for codeine or codeinone also appears to be mostly well-formed and quite distinctive in conformation compared with other AKRs, including the most closely related enzymes in the AKR4 family. The six copies of COR form three pairs of homodimers with *C*_*2*_ point group symmetry. Despite the presence of DTT during crystallization, a disulfide bond between Cys-220 of each protomer was found in each of the three dimer interfaces seen in the asymmetric unit.

### Structural comparisons

The DALI server (21) was used to identify structures in the PDB with the most extensive structural similarity to COR. The top results were the AKR4C9 proposed to function as a promiscuous detoxifying enzyme from *Arabidopsis thaliana* (3H7U, 41% sequence identity, 1.2 Å root mean square deviation (RMSD)), followed by chalcone reductase from *Medicago sativa* (1ZGD, 54% identity, 1.3 Å RMSD). This analysis is consistent with previous surveys of structures from the AKR superfamily, which show that the TIM-barrel fold is universally conserved. Even more distantly related members of the family such as *Homo sapiens* AKR type 3 human hydroxysteroid dehydrogenase (3α-HSD) (1J96, 1.9 Å RMSD) show close structural similarity to COR despite even lower levels of sequence identity of 35%.

**Figure 3:**
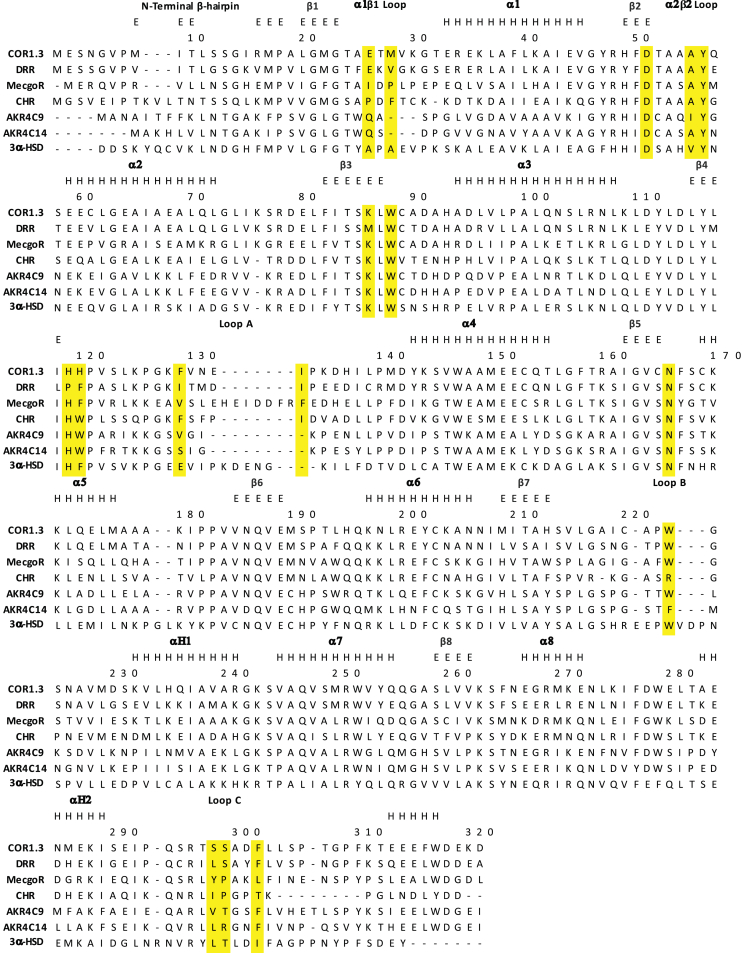
**Multiple sequence alignment of relevant AKRs.** AKR sequences were aligned using Clustal Omega from EMBL-EBI Hinxton (38). Residue numbering corresponding to AKR sequences are shown on the *right*. COR1.3 numbering in steps of ten residues is shown at the top of the alignment. The COR1.3 BIA-binding pocket residues are highlighted in *yellow*. Secondary structure elements were assigned by DSSP (39) where H corresponds to ⍺-helical conformations and E corresponds to β-strand conformation. Abbreviations and accession numbers are as follows: *Papaver sonmiferum*, COR1.3, Q9SQ68.1 (40); *Papaver sonmiferum*, reticuline epimerase (REPI), AKO60181.1; *Erythroxylum coca*, methylecgonine reductase (MecgoR), ADK94763.1 (18); *Medicago sativa*, CHR, AAB41556.1 (41); *Arabidopsis thaliana*, AKR4C9, ABH07515.1 (42); *Oryza sativa Indica Group*, AKR4C14, ACS92968.1; *Homo sapiens*, 3-⍺-HDS, 1J96_A (43).

Regions of structural variation in AKRs are primarily located in the five loops that play major roles in substrate recognition (20). The most notable structural feature of COR is the shifting of the β1α1 loop away from the cofactor binding tunnel, which is made particularly evident when compared with structures of CHR and 3α-HSD (Fig. 2*B*). The unique conformation of this critical loop allows it to stack on top of the apparently more flexible β2α2 loop. The distinctive conformation adopted by the β1α1 loop widens the cofactor binding tunnel and helps to define the putative binding site for the alkaloid substrate, primarily through the side chain and carbonyl group of Glu-26 and the side chain of Met-28. Multiple sequence alignments (Fig. 3) show that Met-28 is unique to COR, whereas Glu-26 is also present in DRR, the only other AKR known to be involved in BIA biosynthesis. Notably, the shifting of the β1α1 loop away from the cofactor binding tunnel does not affect any of the residues involved in NADP(H) binding. The β2α2 loop adopts nearly identical conformations in CHR, AKR4C9, and 3α-HSD, which is not surprising given the critical importance of the two catalytic residues Asp-51 and Tyr-56 at the base of the loop.

Although residues 126–132 of loop A in COR could not be definitively modeled, the weak electron density corresponding to this part of the structure suggests that these residues can adopt multiple conformations, including a conformation that is similar to that seen in CHR and AKR4C9, apart from the clash described below. Notably, in comparison to 3α-HSD, the loop A of COR and CHR is seven residues shorter and that of AKR4C9 is nine residues shorter. COR loop A curves over top and runs down along the side of the substrate-binding pocket, contributing the key catalytic residue His-119. The additional residues in 3α-HSD elongate the loop outward away from the active site, but the portions of the loop proximal to the active site are quite similar to what is seen in COR, CHR, and AKR4C9. Multiple sequence alignments (Fig. 3) show that residues 129–131 are especially variable. These residues are disordered in all six copies of the apo-COR crystal structure and likely form a conformationally dynamic cap or lid on the substrate binding pocket. Structural comparisons of 3α-HSD, CHR, AKR4C9, AKR1C13 (3LN3), and AKR4C14 (6KBL) show that one or two residues out of the three variable positions point into the substrate-binding pocket. In COR, these three residues are Phe-129, Val-130, and Asn-131. However, the distinctive conformation adopted by the β1α1 loop in COR likely blocks Phe-129 from pointing into the substrate-binding pocket.

Loop B contributes to both the cofactor and substrate-binding pockets. Structural conservation of residues 212–219 between COR, CHR, AKR4C9, and 3α-HSD was expected since this region contains residues contributing to cofactor binding. With the exception of CHR, which contains Arg-223 at the equivalent position, the highly conserved Trp-223 residue at the tip of the loop points into the substrate-binding pocket. Although conserved at the primary structure level (Fig. 3), the extent to which Trp-223 penetrates into the active site varies among COR, CHR, AKR4C9, and 3α-HSD. As a result, the precise positioning of Trp-223 residue affects the size and shape of the substrate-binding pocket. Somewhat surprisingly, the longer loop B in 3α-HSD helps to tighten the substrate-binding pocket, whereas the shorter loop B in COR helps to expand the substrate-binding pocket (Fig. S3).

The C-terminus and loop C of COR adopt conformations that are similar to AKR4C9 and to a lesser extent 3α-HSD. Loop C is particularly distinctive in CHR since it is six residues shorter (Fig. 3). Nevertheless, the high degree of structural conservation observed between the substrate-binding pocket residue Phe-302 in COR and equivalent residues in CHR, AKR4C9, and 3α-HSD suggests that these share a conserved functional role in substrate recognition.

### Cofactor binding pocket

**Figure 4:**
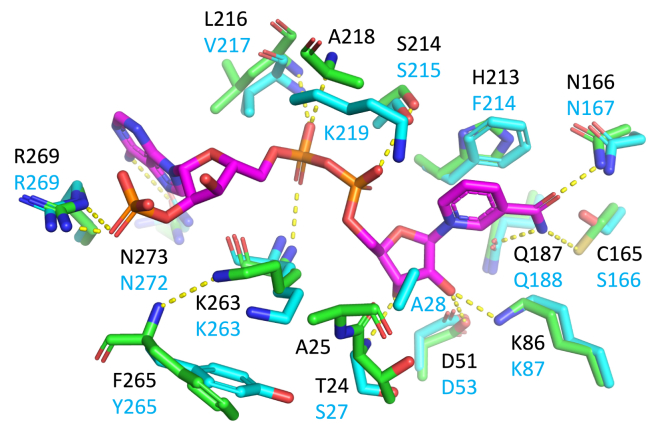
**Model of COR NADP+ binding pocket with superimposed CHR (1ZGD).** COR-NADP^+^-codeine complex model, constructed based on the COR crystal structure, was used to make this stick representation. CHR-NADP^+^ complex (1ZGD) was superimposed over apo-COR structure to place NADP^+^. Side chains of Lys-263, Arg-269, and Phe-265 were moved to remove steric clashes with NADP^+^. *Green* atoms and bonds correspond to COR carbons, *cyan* corresponds to CHR (1ZGD), while *magenta* corresponds to NADP^+^. *Blue* corresponds to nitrogen atoms, *red* to oxygen, and *yellow* to sulfur.

Although NADPH was present at 1 mM during the crystallization of COR, the electron density map indicates that NADPH is not bound to COR in any of the six copies in the asymmetric unit. Packing interactions for this crystal form could favor the apo form of the enzyme, as crystal growth appears to be inhibited at concentrations of NADPH that are higher than 2 mM. Superimposing the structure of the CHR-NADP^+^ (1ZDG) complex onto the structure of apo-COR reveals that the highly conserved cofactor binding pocket seen in all AKRs is also found in COR (Fig. 4). Multiple sequence alignments (Fig. 3) and structural analysis of the AKR superfamily suggest a highly conserved mode of binding (14, 20). The NADP(H)-binding pocket resembles an elongated tunnel formed predominantly by loops B and β1α1. NADPH is expected to bind in an extended anticonformation with the nicotinamide group in the core of the TIM barrel and the adenine moiety more exposed on the surface (19, 20). The “clamp” formed between loop A and the β1α1 loop previously described in CHR (19) is not observed in COR. Instead of closing over the NADP(H) cofactor as seen in CHR, the β1α1 loop is oriented toward the substrate-binding pocket, as described below in more detail. A distinctive feature of the NADP(H)-binding site in COR is the presence of His-213 in a position that is dominated by Tyr and Phe residues in most other members of the AKR superfamily. The side chain of this residue interacts closely with the nicotinamide ring in all AKRs, presumably to help orient it for hydride transfer (20). A network of hydrogen bonds also assists with the positioning of NADP(H) in the tunnel. The carboxamide group on the nicotinamide ring is expected to form hydrogen bonds with the side chains of His-119, His-120, Cys-165, Asn-166, and Gln-187. Additional H-bonds are predicted to be formed between the ribose and the side chains of Asp-51, Lys-86, and Thr-24. The pyrophosphate backbone also forms hydrogen bonds with Ser-214, Ala-218, Leu-216, Lys-263, and Ala-25. Ala-218 in COR corresponds to a Lys residue in CHR that provides an additional hydrogen bond with the pyrophosphate moiety that is not present in COR (19). In COR and other AKRs, the adenosine-monophosphate moiety is also positioned by several hydrogen bonds: Cys-220 with the ribose, Arg-269 and Phe-265 with the adenine, and Asn-273 with the monophosphate. In the apo-COR structure, Cys-220 forms a disulfide bond in the dimer interface with Cys-220 from the adjacent subunit (Fig. S4*A*). Molecular modeling suggests that this disulfide bond does not occur in the NADP(H) complexed form of COR. The Cys-220 disulfide bond and corresponding dimer are further explained below.

### Substrate-binding pocket

**Figure 5:**
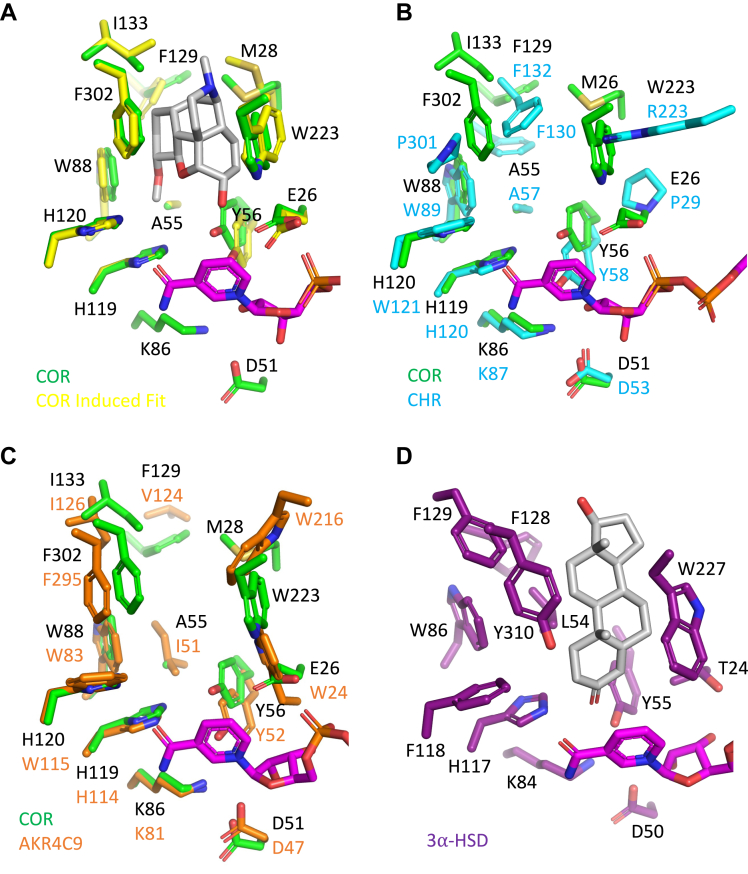
**AKR substrate-binding pockets.***A*, COR in *green* with superimposed NADP^+^ from CHR-NADP^+^ complex (1ZGD) and induced-fit COR in *yellow* with docked codeine in *gray*. *B*, COR in *green* and superimposed CHR in *cyan* (1ZGD). NADP^+^ from CHR (1ZGD) is shown in *magenta*. *C*, COR in *green* and superimposed AKR4C9 in *orange* (3H7U). NADP+ from AKR4C9 (3H7U) is shown in *magenta*. *D*, tertiary 3⍺-HSD-NADP^+^-testosterone (1AFS). Side chains shown in *purple*, testosterone in *gray*, and NADP^+^ in *magenta*. *Blue* corresponds to nitrogen atoms, *red* to oxygen, and *yellow* to sulfur.

The primary substrates in the plant for the reductive reaction catalyzed by COR are codeinone and morphinone, but *in vitro* and in engineered yeast, COR also irreversibly reduces neopinone and neomorphinone, yielding the undesirable by-products neopine and neomorphine (10) (Fig. 1). The substrate-binding pocket of COR is located on top of the TIM barrel near the C-terminal end and is formed predominantly by loops A, B, and C with additional contributions from loops β1α1 and β2α2 (Fig. 5*A*). The residues that form the canonical catalytic tetrad are contributed by the β2α2 loop (Asp-51 and Tyr-56), β3 (Lys-86) and loop A (His-119). As established for other members of the AKR superfamily, Tyr-56 acts as an acid/base catalyst in a proton relay involving His-119 during reduction reactions and involving Lys-86 and Asp-51 during oxidation reactions (14). Superposition of the CHR-NADP^+^ and AKR4C9-NADP^+^ complexes onto COR (Fig. 5, *B* and *C*) shows the structural conservation of the catalytic tetrad with the exception of Tyr-56 of COR, which is shifted 3.3 Å away from the predicted location of NADP^+^ when compared with CHR and AKR4C9. Although Tyr-56 of COR is shifted out of hydrogen bonding distance from Lys-86 and Asp-51, it remains within hydrogen bonding distance of His-119. A likely reason for the shift in position of Tyr-56 is the absence of NADP(H) in the apo-COR structure. A slight shift in the position of the loop and χ_1_ angle of the Tyr-56 side chain would move it to hydrogen bonding distance with Lys-86. The substrate-binding pocket is lined with aromatic (Y56, W88, H119, H120, W223, F129, F302), hydrophobic (M28, I133), and polar residues (E26, D51, K86, D166, S298, S299). Possible conformations of the dynamically disordered region of loop A, including Phe-129, were modeled using Sphinx (22). Induced-fit docking studies of COR (Fig. 5*A*) suggest several likely BIA–side chain interactions. Comparison of COR with docked codeine to 3⍺-HSD-NADP^+^-testosterone demonstrates similar orientation of the substrate ketone/alcohol oxygen to the catalytic tetrad (Fig. 5, *A* and *D*). Docking of codeine places the *N*-methyl moiety in close proximity to the sulfur atom of Met-28. Aromatic sandwiching interactions between Phe-302, codeine and Trp-223 are reminiscent of those seen in other AKRs (23, 24). Other aromatic residues proximal to codeine are Phe-129, Trp-88, and His-120, as well as His-119 and Tyr-56, which are part of the catalytic tetrad. During Induced-fit docking of codeine into COR, Tyr-56 shifts toward a conformation similar to that of CHR (Fig. 5, *A*–*C*). The shifting of Tyr-56 puts it within hydrogen bonding distance of Lys-86 without disrupting interactions with His-119.

### Oligomeric state

The asymmetric unit of this crystal structure of apo-COR reveals three homodimers with *C*_*2*_ rotational symmetry. The dimer interface forms a V-shaped cleft with two active sites facing each other on either side of the cleft (Fig. S4*A*). A single disulfide bond is formed between Cys-220 of each protomer near the bottom of the V-shaped cleft. Size-exclusion chromatography demonstrates that the dimer forms in solution under oxidizing conditions and is destabilized in a reducing environment (1 mM DTT) (Fig. S4*B*). Cys-220 is not conserved in four other reported *Papaver somniferum* COR isoforms, and size-exclusion chromatography failed to detect any dimerization for the COR-B isoform. The lack of conservation of the Cys-220 residue and the reported localization of COR in a reducing environment in the plant suggest that the intermolecular disulfide bond may be the result of purifying and crystallizing COR in an aerobic environment, even though reducing agent was present during purification, and protein samples were stored at –80 °C prior to crystallization. Coagulation of *P. somniferum* latex following herbivory or lancing occurs at least in part due to exposure to the oxidative atmospheric environment and may involve formation of Cys-Cys covalent bonds such as those seen in COR1.3 (25). The biological significance of the homodimer interface adjacent to the putative disulfide bond is also unclear.

### Structure-guided mutagenesis

Site-directed mutagenesis was used to evaluate the potential roles of residues in the putative substrate-binding pocket of COR. The apo-COR crystal structure, models of complexes, and AKR sequence alignments were closely consulted and cross-referenced to design a series of 17 mutants exploring the role of key residues with respect to catalysis, binding, and substrate recognition. In general, cobalt affinity-purified wild-type and mutant COR protein preparations were quite comparable, although two mutants (H119F, F302A) produced lower yields and purity (Fig. S5). For these two mutants, the formation of insoluble inclusion bodies in *Escherichia coli* was observed, suggesting that the mutations lead to protein misfolding. Consistent with the observation of disulfide-linked dimers in the COR crystal structure despite the use of a reducing agent during purification, SDS-PAGE analysis under reducing conditions revealed minor bands at twice the expected molecular weight of monomeric COR. Wild-type and mutant COR proteins preparations were characterized using “standard” assays to accurately quantify the major reduction and oxidation activities under initial-rate product formation conditions, and “extended” assays were used to detect the minor neopinone reductase activity as described previously (10). For the H119F and F302A mutants, assays were not corrected for impurities in the protein samples, which exhibited approximately half the purity of other mutants. Nevertheless, the detected activity in the assays was less than 15 and 2%, respectively, compared with wild-type COR1.3 suggesting that mutations of these residues significantly impaired enzyme function beyond the reduced activity attributable to reduced protein purity.

### “Standard” assay

**Figure 6:**
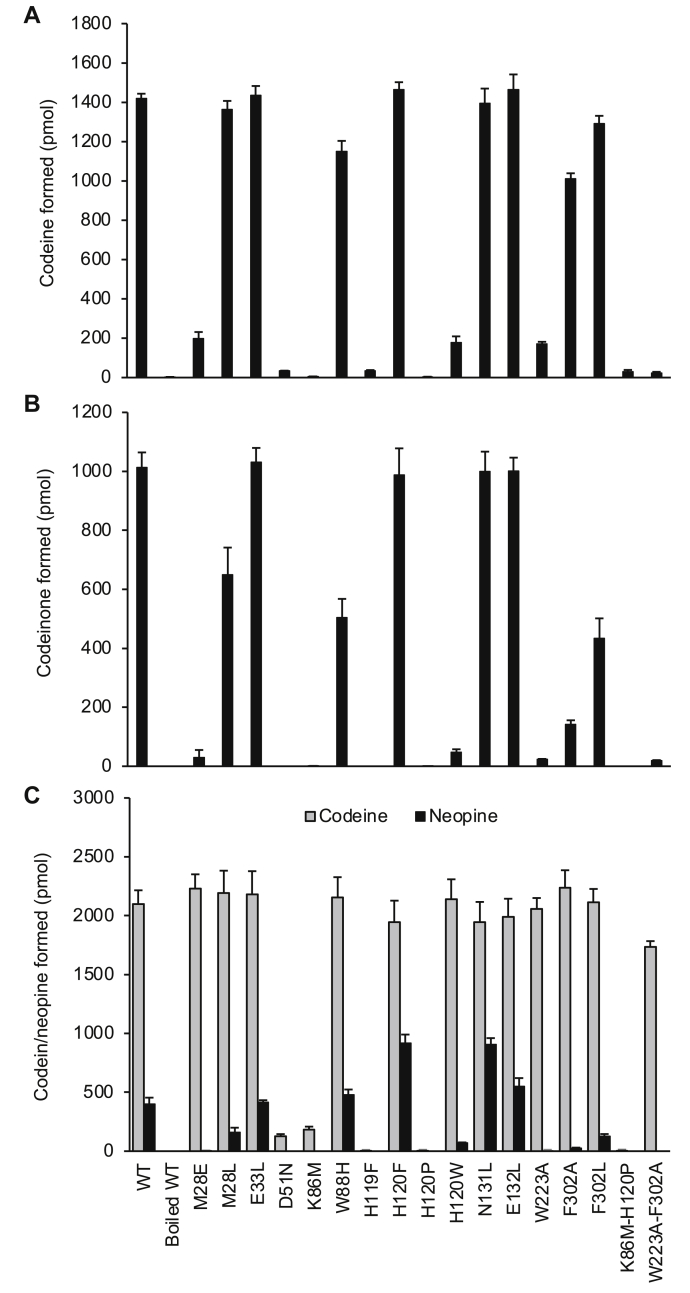
**Activity of COR mutants.***A*, reductive forward direction assays contained 50 μM codeinone/neopinone (3:2 equilibrium) and 1 mM NADPH and were conducted at pH 6.8. *B*, oxidative reverse direction assays contained 75 μM codeine and 1 mM NADP^+^ and were conducted at pH 9.0. Assays contained 0.2 μg purified recombinant protein and 100 mM bis-tris propane buffer in a total volume of 50 μl, and were incubated at 30 °C for 10 min. Reported values of codeinone formed include neopinone derived from spontaneous codeinone isomerization. *C*, activity of COR mutants in extended forward assays. Formation of codeine (*black bars*) and neopine (*gray bars*) in 180 min assays containing 2 μg purified recombinant protein, 100 μM codeinone/neopinone (3:2 equilibrium) and 1 mM NADPH in 100 mM bis-tris propane buffer at pH 6.8 in a total volume of 50 μl. Error bars represent the standard deviation of three independent replicates.

All mutations of the canonical catalytic tetrad (D51N, K86M, and H119F) resulted in a complete loss of detectable oxidative activity and only trace levels of reductive activity (Fig. 6, *A* and *B*). Eight residues potentially involved in substrate binding and recognition were selected for study based on the lack of sequence conservation across the plant AKRs including COR, CHR, MecgoR, and DRR (Fig. 3). Of these, substitutions at five positions, which line the sides of the putative substrate binding pocket, were shown to affect COR activity (Met-28, Trp-88, His-120, Trp-223, and Phe-302). The remaining three residues that were selected by the same reasoning did not alter COR activity and are located in loop A lining the top of the substrate-binding pocket (Asn-131 and Glu-132) or NADP(H)-binding pocket (Glu-33).

The substitution of residues lining the putative substrate binding pocket shows effects that are consistent with the importance of the wild-type residues for positioning substrates in productive positions allowing for catalysis. Because Trp-223 and Phe-302 residues line opposite sides of the binding pocket and may cooperatively hold the aromatic A ring in place, their function was examined further in a double mutant. Although the reductive activity of W223A-F302A was lower than in either single mutant, oxidative activity was not lower than that of the W223A single mutant. The general observation that BIA-binding pocket mutants decreased oxidative activity more so than reductive activity suggests that the effects of the substitutions on positioning the substrate for oxidation, in which proton transfer is to the structurally restricted Tyr-56 acceptor, may be greater than the effects on the positioning of the substrate for reduction, in which hydride transfer occurs from noncovalently bound and perhaps more adaptable NADPH.

The binding-pocket mutations can be classified into three groups. The first group includes the mutations that did not alter COR activity and consists of four mutants of residues lining the substrate binding site (E33L, H120F, N131L, and E132L). The second group includes the mutations that altered but did not abolish COR activity. This group consisted of seven mutants targeting five residues lining the substrate binding site (M28E, M28L, W88H, H120W, W223A, F302A, and F302L). All the group two mutants decreased COR oxidative activity more than reductive activity. No mutants reduced reductive activity more than oxidative activity. The third group are the mutants that had undetectable oxidative and nearly undetectable reductive COR activity. These consist of the three catalytic tetrad mutants (D51N, K86M, H119F) and one mutant of a residue lining the substrate-binding site (H120P).

### “Extended” assay

All mutants described above were also characterized using extended incubation times, which do not reflect enzyme activity as typically measured under the initial-rate product formation conditions used for the wild-type enzyme or the more active mutants. With extended incubation times, wild-type COR revealed a modest neopinone reductase activity (Fig. 6*C*). In general, the three groups of mutants described above showed trends in the extended assays, which are comparable to those in the standard assays. Group one mutants (unaffected; E33L, H120F, N131L, E132L) reduced codeinone/neopinone to codeine at levels indistinguishable from COR wild-type. E33L was also equivalent to wild-type in terms of neopine production. However, H120F, N131L, and to a lesser extent E132L produced substantially more neopine (up to 300% of wild-type). All but one of the group two mutants produced the same amount of codeine as wild-type COR but little to no neopine. The exception, W88H, was equivalent to wild-type COR in all respects including neopine formation. As expected, group three mutants formed very little product despite the extended incubation times. D51N and K86M produced small quantities of codeine (5–10% of wild-type COR), whereas all group three mutants produced no detectable amounts of neopine.

## Discussion

### BIA-binding pocket

Prior to the COR structure reported herein, the most closely related AKR structure with respect to sequence identity was the isoflavonoid biosynthesis enzyme chalcone reductase (CHR). A previously reported homology model built using the CHR-NADP^+^ complex structure (19) as a template suggested some of the features in COR responsible for BIA recognition. COR homology models also helped infer structure–function relationships of four residues (Trp-279, Lys-41, Phe-29, and Ala-25) identified through sequence analysis and mutagenesis studies of COR isoforms in *P. somniferum* (10). With one exception, the predictions made in that study still hold in light of the apo-COR structure. Ala-25 lies in a different location than expected based on the CHR structure due to the substantial change in location of the β1α1 loop toward the BIA-binding pocket, which places the sidechains of Met-28 and Glu-26 into the BIA-binding pocket. Considering the variation of β1α1 loop conformation in related CHR and 3-α-HDS (Fig. 2*B*), it is possible that the β1α1 loop can adopt several conformations owing to an inherent flexibility. However, mutagenesis of Met-28 suggests that the observed conformation of the β1α1 loop in apo-COR has biological function. Mutagenesis studies reported herein demonstrate for the first time the importance of unexpected residues lining the distinctive structure of the BIA-binding pocket, which could not be predicted based on homology modeling from templates with different structures of the β1α1 loop. Most notably, changing the side chains of the β1α1 Met-28 loop residue had a dramatic effect on activity. Changes to Trp-88, His-120, Trp-223, and Tyr-302 lead to substantial changes in the activity of COR. Mutations in residues Arg-131 and Glu-132 had minimal effects on activity, suggesting that these residues in a region of loop A lining the top of the substrate-binding pocket might not be important for substrate position and catalysis. The functional contributions of loop A to defining BIA substrate recognition and catalysis are less clear due to the dynamic disorder present in the loop and lack of electron density from the crystallographic analysis. Previous mutagenesis work (10) showed that changes to the side chain of the loop A residue Phe-129 affect neopine formation. Given that Phe-129 is conserved across all but one COR isoform (Leu-129 in COR1.2) and in these previous results, loop A is almost certainly involved in BIA binding. Given that enzyme assays were conducted under maximal product formation conditions and not nearer to reported *K*_m_ values (10), there remains some ambiguity as to whether the effect on activity is the result of perturbed binding, turnover, or a combination of both.

Our mutagenesis experiments show particularly intriguing changes in activity resulting from specific substitutions at two positions. The M28E mutant shows significant decreases in both oxidative and reductive activity, whereas the M28L mutant shows a smaller decrease and only in the oxidation reaction. Both substitutions decrease neopine formation in extended assays. Apparent discrepancies between standard and extended assay results can be reconciled by considering the former as an accurate determination of enzyme specific activity and the latter as a measure of an activity endpoint controlled by other factors (*e.g.*, product inhibition), which can be achieved even by mutants with reduced function. The different functional consequences of the side chain substitutions may indicate that the charged side chain of Glu destabilizes the binding of the hydrophobic BIA substrate more so than the wild-type Met or the hydrophobic Leu. Since M28L affected the oxidation reaction more than the reduction reaction, Leu may negatively impact the catalysis of the oxidation of codeine to a greater extent than the reduction of codeinone. The modeled COR loop A, which is similar to homology models, places Phe-129 behind Met-28 and likely too far to directly contact the BIA substrate. However, previous mutagenesis studies showed that the F129L mutation in COR-B decreases oxidation of codeine and increases neopine production (10). Our structure suggests an explanation of this effect through an indirect mechanism. Changes in the side chain at position 129 are expected to alter the position of the side chain of Met-28, thereby modifying the size and shape of the substrate-binding pocket. Phe-129 also forms aromatic interactions with Trp-88, which is also part of the substrate-binding pocket. A third effect is suggested by induced-fit docking studies, which show how a modest shift of the β1α1 loop could allow Phe-129 to interact directly with the BIA *N*-methyl group.

**Figure 7:**
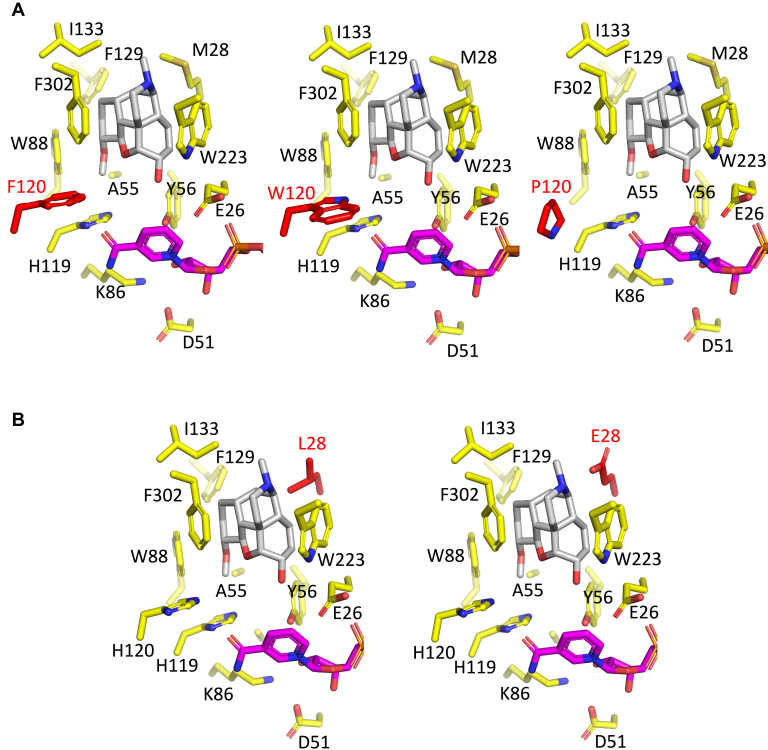
**BIA-binding pocket models for COR mutants.** Induced-fit COR-NADP^+^-codeine model with side chains shown in *yellow*, codeine in *gray*, and NADP^+^ in *magenta*. *Red* side chains correspond to substituted residue. *Blue* corresponds to nitrogen atoms, *red* to oxygen, and *yellow* to sulfur. *A*, COR H120F, H120W, and H120P mutants. *B*, COR M28L and M28E mutants.

Our structure also suggests for the first time how aromatic interactions between His-119 and His-120 may be important in correctly orienting and activating His-119 for proton relay with Tyr-56 and bulk water (Fig. 7*A*). Substitution of His-120 with three different residues shows vastly different effects on COR activity. H120P abolishes COR activity. As the proline substitution disrupts aromatic stacking with His-119 and may also change the backbone conformation due to additional φ and ϕ torsion angle restrictions, we hypothesize that the H120P mutation moves His-119 out of range for efficient proton transfer. In contrast, H120F, which mimics the DRR active site, showed no effect on COR activity, because the aromatic Phe side chain does not disrupt stacking interactions with His-119 and resembles His enough to maintain interactions with the BIA substrate. The lack of negative consequences resulting from the substitution of His-120 with a residue that lacks hydrogen bonding capabilities suggests other modes of interaction. H120W, which mimics the CHR active site, substantially decreased COR oxidative, and reductive activity. Although aromatic stacking with His-119 is not disrupted, the larger bicyclic side chain of Trp likely reduces the size of the BIA-binding pocket enough to disrupt the binding of codeine and codeinone.

### Neopine production

The substrates for the reduction reaction catalyzed by COR, codeinone, and neopinone spontaneously interconvert through a slow isomerization reaction. At physiologically relevant temperatures *in vitro*, strong COR activity (*e.g.*, COR-B) converts most of the neopinone produced from thebaine by T6ODM to neopine before the neopinone can isomerize to codeinone. Under the practical conditions used for the production of codeine using metabolically engineered organisms, COR has been shown to irreversibly reduce neopinone to neopine (and neomorphinone to neomorphine in the mirror pathway) (10), which compromises the efficiency of the process and generates a substantial amount of an undesirable waste product (8, 23). The lack of significant levels of neopine in *P. somniferum* indicates a mechanism to limit neopine formation in the plant. This mechanism was recently attributed to the enzyme neopinone isomerase (NISO), which has been shown to catalyze the reversible isomerization of neopinone and codeinone (12). The discovery of NISO as an enzymatic catalyst to increase the rate of interconversion between neopinone and codeinone, as well as between neomorphinone and morphinone (Fig. 1), also provides a practical molecular tool to limit neopine and neomorphine accumulation in engineered yeast (12). To further assist with directing metabolic flux away from the less desirable side products, COR mutants exhibiting decreased neopine formation compared to wild-type would be of additional benefit.

With this goal in mind, the M28E mutant was semirationally designed to improve the binding of codeinone over neopinone, assuming that the positively charged amino groups in codeinone and neopinone may interact with the negatively charged carboxylate group of the Glu-28 side chain in different ways (Fig. 7*B*). Indeed, extended assays reveal that the M28E mutant enzyme produces as much codeine as wild-type COR, whereas neopine formation is greatly decreased (less than <1% of wild-type), showing a highly desirable effect. Experimental information from crystal structures of COR in complex with codeinone and neopinone would help to refine and test these predictions. A few other mutants (M28L, H120W, W223A, F302A, and F302L) also showed a preference for codeine formation over neopine formation (Fig. 6*C*). Conversely, the H120F mutant increased neopine production in extended assays in a manner comparable to previous mutagenesis efforts (10). Although the COR mutant results reported herein are from *in vitro* assays only, the long incubation times in the extended assays provide a good approximation of the conditions in heterologous biosystems such as engineered yeast and thus strongly suggest that engineering COR is a worthwhile approach to reducing neopine formation further, especially in combination with the effects of NISO activity.

### Insights into DRR, the AKR domain found in REPI

**Figure 8:**
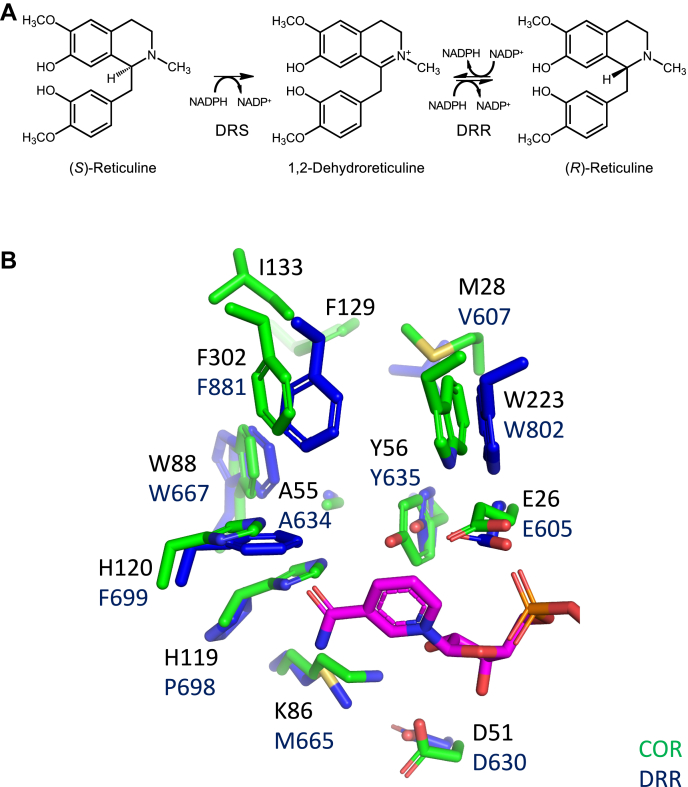
**DRR homology model. DRR homology model.***A*, the two-step stereochemical conversion catalyzed by REPI of (*S*)-reticuline to (*R*)-reticuline, which is converted *via* several enzymes to morphine. (*S*)-Reticuline is converted to 1,2-dehydroreticuline by DRS, and 1,2-dehydroreticuline is stereospecifically reduced to (*R*)-reticuline by DRR. *B*, superimposed NADP^+^ from CHR (1ZGD) is shown in *magenta*, DRR side chains are shown in *blue* with REPI numbering, and COR side chains are shown in *green*. *Blue* corresponds to nitrogen atoms, *red* to oxygen, and *yellow* to sulfur.

The elucidation of the COR crystal structure allows for the first time the generation of more reliable homology models for 1,2-dehydroreticuline reductase (DRR; 72% amino acid identity) (Fig. 8), the only other known AKR involved in BIA biosynthesis. DRR is the AKR domain of the reticuline epimerase (REPI) fusion enzyme, in which a cytochrome P450 domain first oxidizes (*S*)-reticuline to 1,2-dehydroreticuline, and then DRR catalyzes a stereospecific reduction of the C=N double bond in 1,2-dehydroreticuline to (*R*)-reticuline (5, 24) (Fig. 8*A*). Despite a high level of sequence identity and a close phylogenetic relationship, sequence alignments of COR and DRR reveal several nonconserved residues in the canonical catalytic tetrad seen in COR. With respect to functionally characterized AKRs, several unique substitutions are observed in DRR including the replacement of His-119 with Pro and the replacement of Lys-86 with Met (numbering as in COR) (Fig. 8*B*). The lack of titratable protons in the active site side chains Pro-698 and Met-665 (corresponding to His-119 and Lys-86 in COR respectively) indicates that the proton transfer steps in the canonical AKR mechanism cannot occur in DRR.

Comparison of DRR with COR and members of the steroid reductase AKR subfamily, including the extensively investigated enzyme AKR1D1 (Human steroid 5β-Reductase), which catalyzes the stereospecific NADPH-dependent reduction of the C4-C5 double bond of bile acid intermediates and steroid hormones, suggests that DRR may employ a partially analogous catalytic mechanism. The reduction of a carbon–carbon double bond by AKR1D1 is accompanied by a characteristic change in the canonical catalytic tetrad relative to other members of the AKR superfamily. Glu takes the place of the almost universally conserved His residue (*e.g.*, His-120 in COR) (14) and two complementary functional consequences have been proposed for the substitution. By donating a hydrogen bond to the steroid reactive oxygen atom, the protonated side chain of Glu is proposed to create a “superacid” oxyanion hole. In combination with the protonated general acid catalyst Tyr residue, this promotes enolization of the steroid ketone and hydride transfer from NADPH to the adjacent 5β carbon. The second role for Glu is proposed to be primarily steric in nature—the less bulky side chain allows the steroid substrate to penetrate deeper into the active site such that the 5β carbon is better positioned to accept the hydride from NADPH. Support for these mechanisms is provided by a series of complex crystal structures, and mutagenesis results in which the single amino acid substitutions (H120E in AKR1D1, H117E or H117A in AKR1C9) readily interconvert the substrate specificities of 5β- and 3β-reductase AKRs (26, 27, 28). Given that the equivalent residue in DRR is a nontitratable Pro-698 rather than the typical His or Glu residue typically found in steroid 3β- and 5β-Reductase AKRs, we hypothesize that the second function (*i.e*., alleviation of steric hinderance) may be especially important in DRR. In addition, the presence of another residue in DRR (Glu-605) that is predicted to be close to the highly conserved Tyr-635 residue (albeit on the opposite face) suggests that Glu-605 may adopt a role similar to the catalytic function of Glu-120 in AKR1D1.

The COR structure, mutagenesis work, and comparative analysis presented here substantially improve our understanding of AKRs with respect to the biosynthesis of important medicinal compounds codeine and morphine. In addition to clarifying the significance of molecular evolution events in the COR/DRR lineage and highlighting the possibility of analogous catalytic mechanisms having evolved independently in two very distinct lineages, the deeper understanding of structure–function relationships in COR should lead to further improvements in the performance of microbial BIA biosynthesis systems. Although still not commercially viable, microbial biosynthesis systems are quickly gaining ground on the traditional agricultural methods of obtaining these medicines and will one day lead to a pharmaceutical production process, which is more environmentally friendly, globally equitable, and easier to secure from illicit diversion.

## Experimental procedures

### Chemicals

Chemicals and reagents used for *in vitro* enzyme assays were obtained as described previously (10). Media components were purchased from Sigma-Aldrich or BioShop Canada. All controlled substances were acquired and used with appropriate government approval.

### Expression and purification

For crystallographic studies, the COR1.3 isoform (AAF13738) was recombinantly expressed in Rosetta 2 *E. coli* cells transformed with the pET47b-COR1.3 expression vector. Starter cultures were grown overnight in 50 ml Luria-Bertani (Miller) broth supplemented with 30 mg/L kanamycin and 35 mg/L chloramphenicol (LBKC) at either 25 or 30 °C with shaking at 170 rpm to an OD_595_ value of ∼0.4, and subsequently used to inoculate six 1-L cultures using LBKC broth. Cultures were grown at 37 °C to an OD_595_ value of 0.5–0.6 and cooled to 18 °C for 30 min. Isopropyl β-D-1-thiogalactopyranoside was added to a final concentration of 1 mM to induce recombinant protein expression, and cultures were incubated at 18 °C for 18–20 h. Cells were then harvested by centrifugation, and cell pellets were resuspended in lysis buffer (50 mM sodium phosphate pH 8.0, 10 mM imidazole, 300 mM NaCl, 15% [v/v] glycerol). Resuspended pellets stored at –80 °C were thawed and lysed by sonication in the presence of lysozyme and DNase, and cell debris was subsequently removed by centrifugation at 4 °C. Lysate was loaded onto a 1-mL HisTrap HP column (GE Healthcare) and eluted using an imidazole gradient on a BioLogic DuoFlow FPLC. Pooled fractions were dialyzed overnight against IEC buffer (20 mM Tris-HCl, pH 8; 0.25 mM EDTA; 1 mM dithiothreitol (DTT); 30 mM NaCl), loaded onto a 5 ml HiTrap Q HP column (GE Healthcare), and proteins were eluted using an optimized salt gradient on a BioLogic DuoFlow FPLC. Pooled fractions were diluted 2-fold in proteolysis buffer (50 mM Bis-Tris-HCl, pH 7.0; 150 mM NaCl; 1 mM EDTA; 1 mM DTT; degassed water) overnight followed by PreScission protease (Thermo Fisher) digestion to cleave off the polyhistidine tag. GST-tagged protease was removed by running the protein sample through Glutathione Sepharose 4B (GE Healthcare) resin. Cleaved protein was dialyzed overnight against the final buffer (20 mM Tris-HCl, pH 8.0; 30 mM NaCl; 2 mM DTT; 0.25 mM EDTA) and spin concentrated to a final concentration of 5 mg ml^−1^. Concentrated protein was flash-frozen in liquid nitrogen and stored at –80 °C. For enzyme assays, COR1.3 expression and purification were carried out as described previously (10). Protein concentrations were determined on a Thermo Fisher nanodrop 1000 spectrophotometer using the theoretical extinction coefficient (29) based on absorbance at 280 nm.

### Crystallization and X-ray diffraction collection

The COR1.3 isoform was crystallized at 5 mg/ml in the presence of 1 mM NADPH and 1 mM codeine in 24% (v/v) polyethylene glycol 3350, 0.35 M sodium chloride, 8% glycerol, 2 mM DTT, and buffered at pH 8.0 with 0.1 M Tris-HCl *via* hanging drop vapor diffusion at room temperature. Single crystals (∼0.12 × 0.05 × 0.02 mm) were harvested using polymer loops (MiTeGen) and flash-frozen in liquid nitrogen. Crystals were stored in liquid nitrogen until mounted in a nitrogen gas stream at 100 K for diffraction data collection. X-ray diffraction data was measured at the Stanford Synchrotron Radiation Laboratory (SSRL) beamline 12-2 using radiation at a wavelength of 0.98 Å and a Pilatus 6M pixel array detector (Dectris). HKL-3000 and Scalepack (30) were used for data processing and phases were calculated by molecular replacement using the structure of chalcone reductase (54% sequence identity, 1ZGD) as a search model with PHASER, as implemented in PHENIX (31). Refinement was performed with REFMAC and PHENIX, and COOT was used for model building (32). The quality of geometric parameters in the model was assessed using Molprobity (33).

### Modeling the structures of COR complexes

A model of COR complexed with NADPH and codeinone was built by superimposing the structure of the CHR-NADP+ complex (1ZGD) onto the structure of the COR apoenzyme. Due to the high level of sequence and structural conservation of residues in the AKR NADP(H)-binding pocket (13, 14), NADPH binding is expected to be very similar in COR. Using CHR and xylose reductase (1K8C) for reference, the side chain conformations of three residues (K263, R269, and F265) were adjusted slightly to prevent steric clashes with the bound conformation of NADPH. The unmodeled residues 126–132 in loop A from the calculated COR structure were modeled using the Sphinx server (22) A range of stereochemically reasonable conformations of the β1α1 loop was also generated using Sphinx to show that a slight change in backbone torsion angles allows for a slight widening of the NADP(H)-binding pocket to accommodate the binding of alkaloid substrates. The COR substrates codeine and codeinone were docked into the modeled active site using Schrodinger Maestro Glide Extra Precision (34) and Prime Induced-fit modules (35). The reactive oxygen atom of the ligand was constrained to 3 Å from the 4-pro-R hydrogen of the nicotinamide ring of NADPH and 3 Å from the oxygen atom of Tyr-56 side chain. The DRR homology model was prepared with MODELLER (36) using COR as a template.

### Mutagenesis

Site-directed mutagenesis was performed using the pET47b-COR1.3 plasmid described previously (10) as the template. Targeted codons were altered by PCR site-directed mutagenesis using Q5 High-Fidelity DNA polymerase (New England Biolabs) and oligonucleotide primers (Integrated DNA Technologies) with point substitutions (37) (Table S1). All constructs were verified by dideoxynucleotide chain-terminator sequencing.

### *In vitro* enzyme assays

Reductive (physiologically forward) and oxidative (physiologically reverse) reactions were carried out as described previously (10) with minor modifications. Standard reductive assays contained 0.2 μg purified recombinant protein, 50 μM codeinone/neopinone (3:2 equilibrium), 1 mM NADPH, and 100 mM bis-tris propane-HCl buffer, pH 6.8, in a total volume of 50 μl. Standard oxidative assay conditions were identical except for the buffer pH (9.0) and the use of 75 μM codeine and 1 mM NADP^+^ as substrates. Reaction mixtures were incubated at 30 °C for 10 min and terminated by the addition of four volumes of acetonitrile. Extended reductive assay conditions were identical to the standard assays described above except for incubation times of 180 min and the use of 100 μM codeinone/neopinone and 2 μg purified recombinant protein. For all enzyme assays, denatured (boiled for 15 min) recombinant COR1.3 protein served as the negative control. Enzyme assays were independently repeated in triplicate and analyzed by LC-MS as described previously (10). Analytes were quantified using a five-point standard curve for each authentic alkaloid.

### Size exclusion chromatography

Size-exclusion chromatography was used to investigate the oligomeric state of COR1.3. For oxidizing conditions, urified COR1.3 (40 μl of a 0.5 mg/ml solution) was diluted in running buffer and incubated overnight at room temperature and subsequently loaded at a concentration of 0.1 ml/min onto a 2.4 ml Superdex 200 column (GE Healthcare) equilibrated in running buffer (25 mM Tris-HCl, pH 7.5, 150 mM KCl, 0.5 mM EDTA). The same procedure was used for reducing conditions, except for the addition of 1 mM DTT to the running buffer (25 mM Tris-HCl, pH 7.5, 150 mM KCl, 0.5 mM EDTA, 1 mM DTT). Molecular weight was interpolated from peak retention time using a calibration curve consisting of aldolase (158 kDa), bovine serum albumin (66 kDa), carbonic anhydrase (29 kDa), and cytochrome c (12.4 kDa). Blue dextran (2000 kDa) was used to determine the void volume.

### Data availability

All data that are part of this study are included within this article. The structure factors and coordinates for the apo-COR crystal structure have been deposited with the Protein Data Bank under accession code 7MBF.

## Supporting information

This article contains supporting information.

## Conflict of interest

The authors declare that they have no conflicts of interest with the contents of this article.
