# Supplementary material for: Structural studies of codeinone reductase reveal novel insights into aldo-keto reductase function in benzylisoquinoline alkaloid biosynthesis
Source: J Biol Chem. 2021 Sep 20;297(4):101211. doi: 10.1016/j.jbc.2021.101211 (PMC8524200; doi:10.1016/j.jbc.2021.101211)
Supplement: Supplementary file 3 — Figures S1–S5 and Table S1 [file mmc3.pdf]

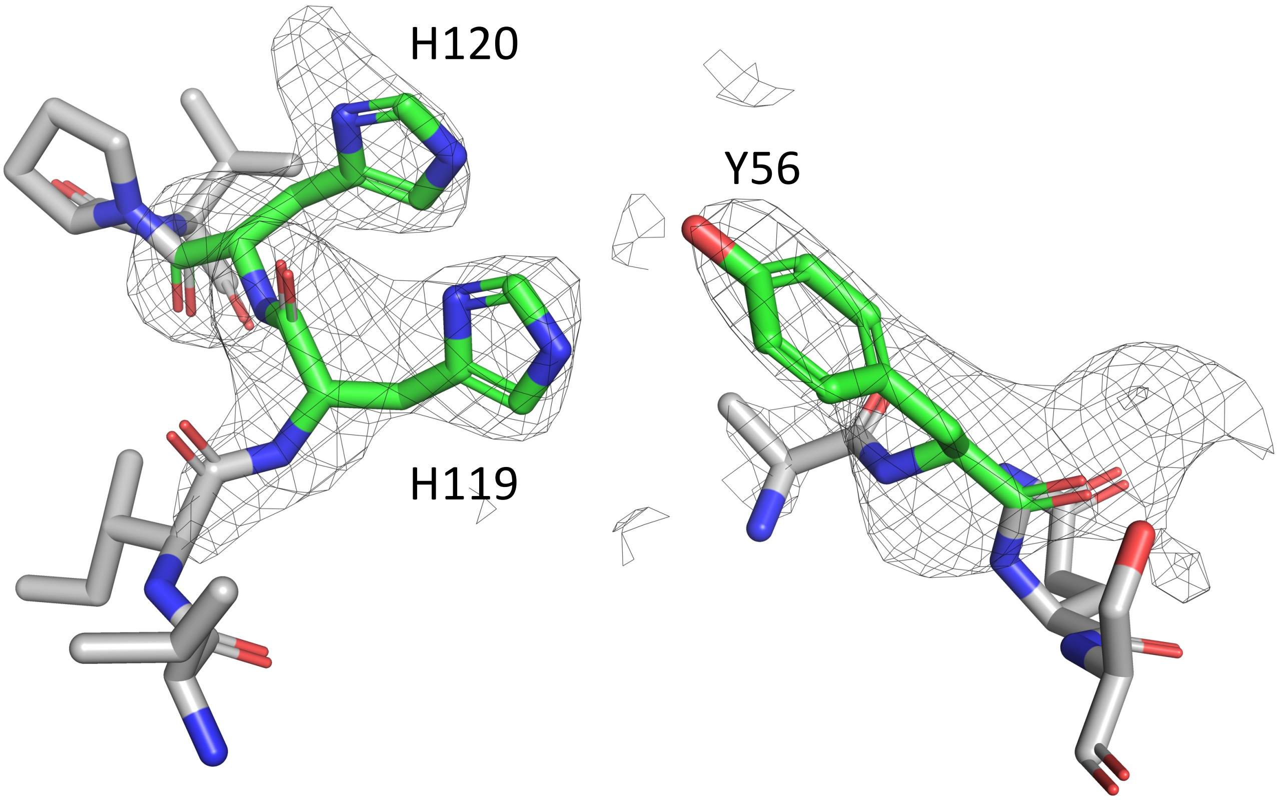

Figure S1. Simulated annealed $|F_o|$ - $|F_c|$ omit map at 3σ for Tyr-56, His-119, and His-120. A) COR residues Tyr-56, His-119, and His-120 are shown in green and neighbouring residues in grey. The $|F_o|$ - $|F_c|$ omit map at 3σ is represented as grey mesh. Start and end temperature for simulated annealing were 5000K and 300K. B) Movie rotating around the y-axis of panel A.

Figure S2. Movie highlighting several key structural features of COR. Rotations were chosen to provide additional context to the two-dimensional views shown in other figures. The crystal structures of CHR (1ZGD) in cyan and 3-a-HSD (1J96) in orange have been superimposed onto the structure of apo0COR. Models of NADP+ and codeine bound to COR are also shown.

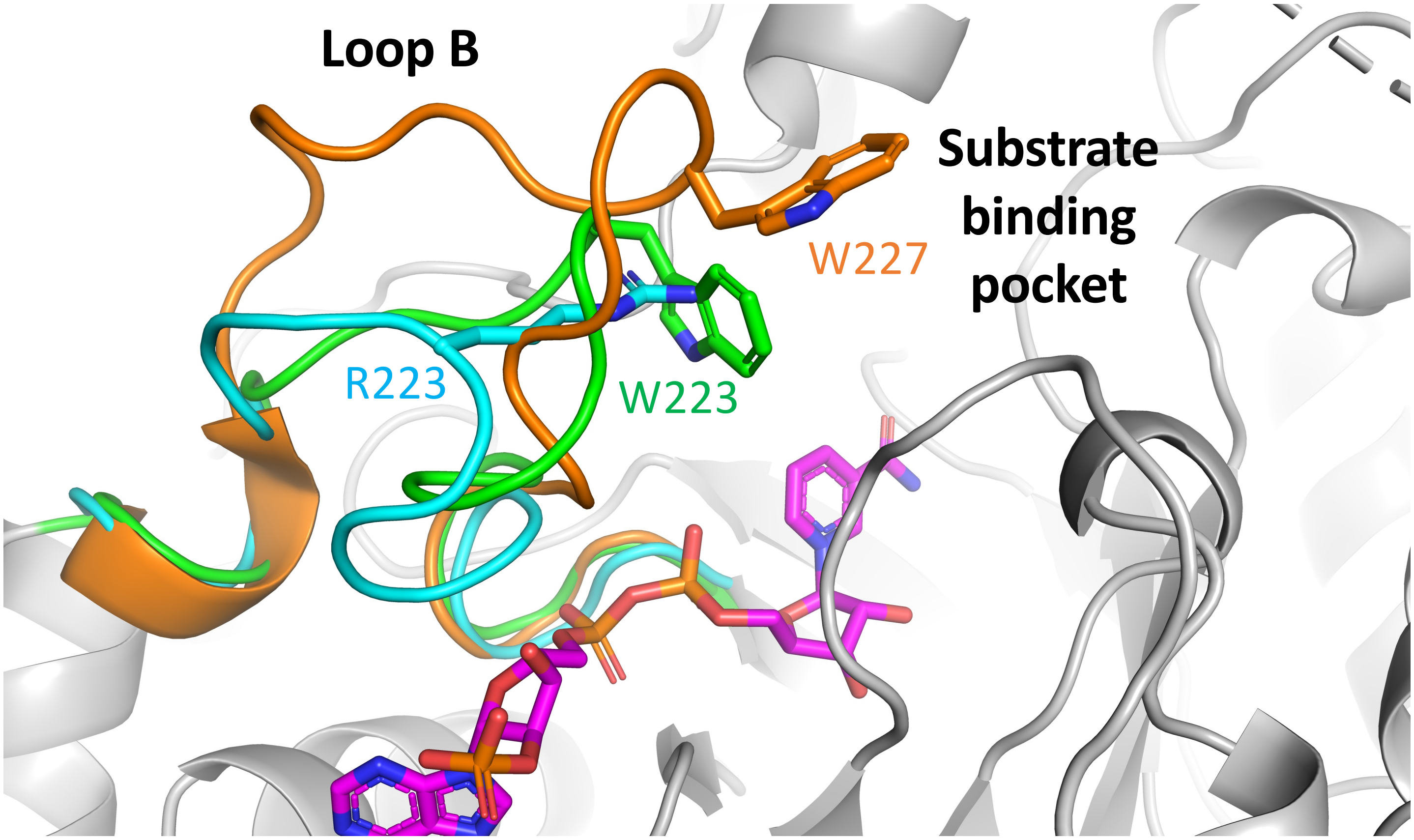

Figure S3. Loop B conformation of apo-COR1.3, CHR, and 3-a-HDS. Superposition of loop B from CHR (1ZGD) in cyan and 3-a-HDS (1J96) in orange on apo-COR1.3 in grey with loop B shown in green. NADP$^+$ from superimposed CHR (1ZGD) is shown in magenta. Blue corresponds to nitrogen atoms, red to oxygen, and yellow to sulfur

A

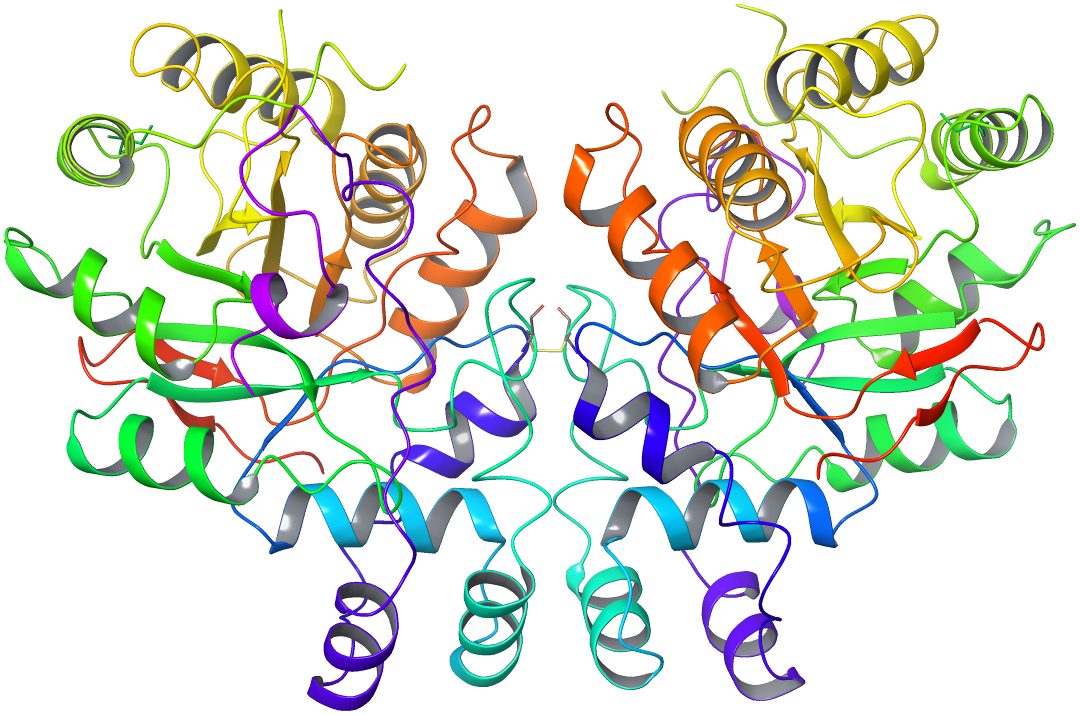

B

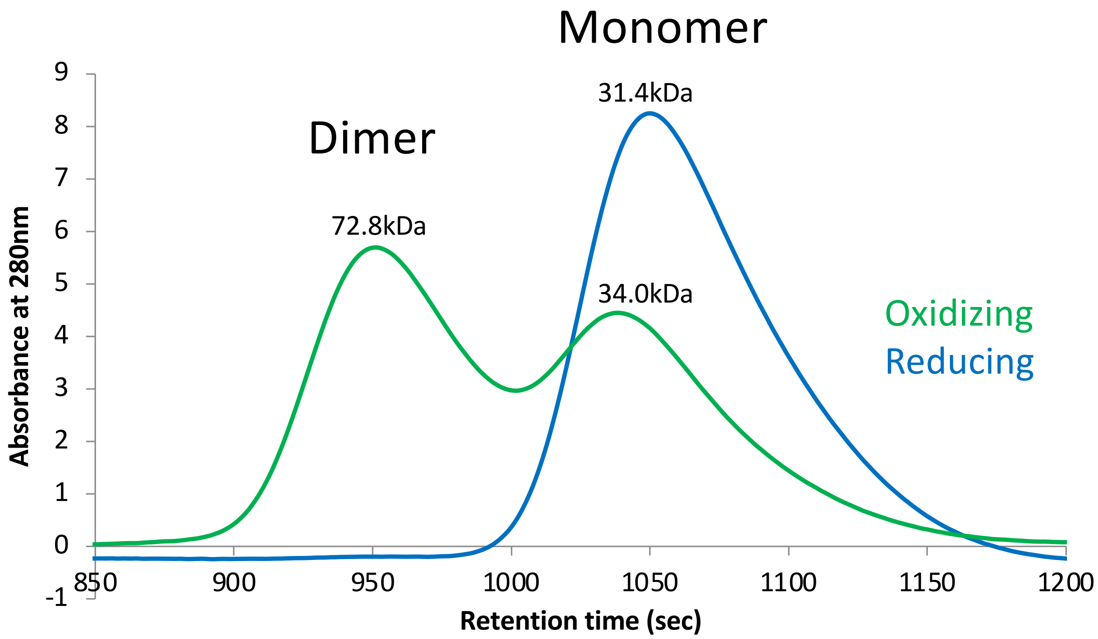

Figure S4. COR1.3 homodimer.
A) Protomers A and D are shown forming one of three homodimers seen in the asymmetric unit. Such a homodimer is consistent with observations made using size-exclusion chromatography under oxidizing conditions. Polypeptide chains are shown as helices and sheets color-coded from N- (blue) to C- (red) terminus. The covalent Cys-220:Cys-220 bridge of unknown biological significance at the base of the V-shaped cleft is shown in stick representation. B) Size-exclusion chromatography of COR1.3 in oxidizing conditions (green) and reducing conditions, 1 mM DTT (blue). Calculated molecular weights are shown above corresponding peaks. Molecular weights were calculated from a standard curve.

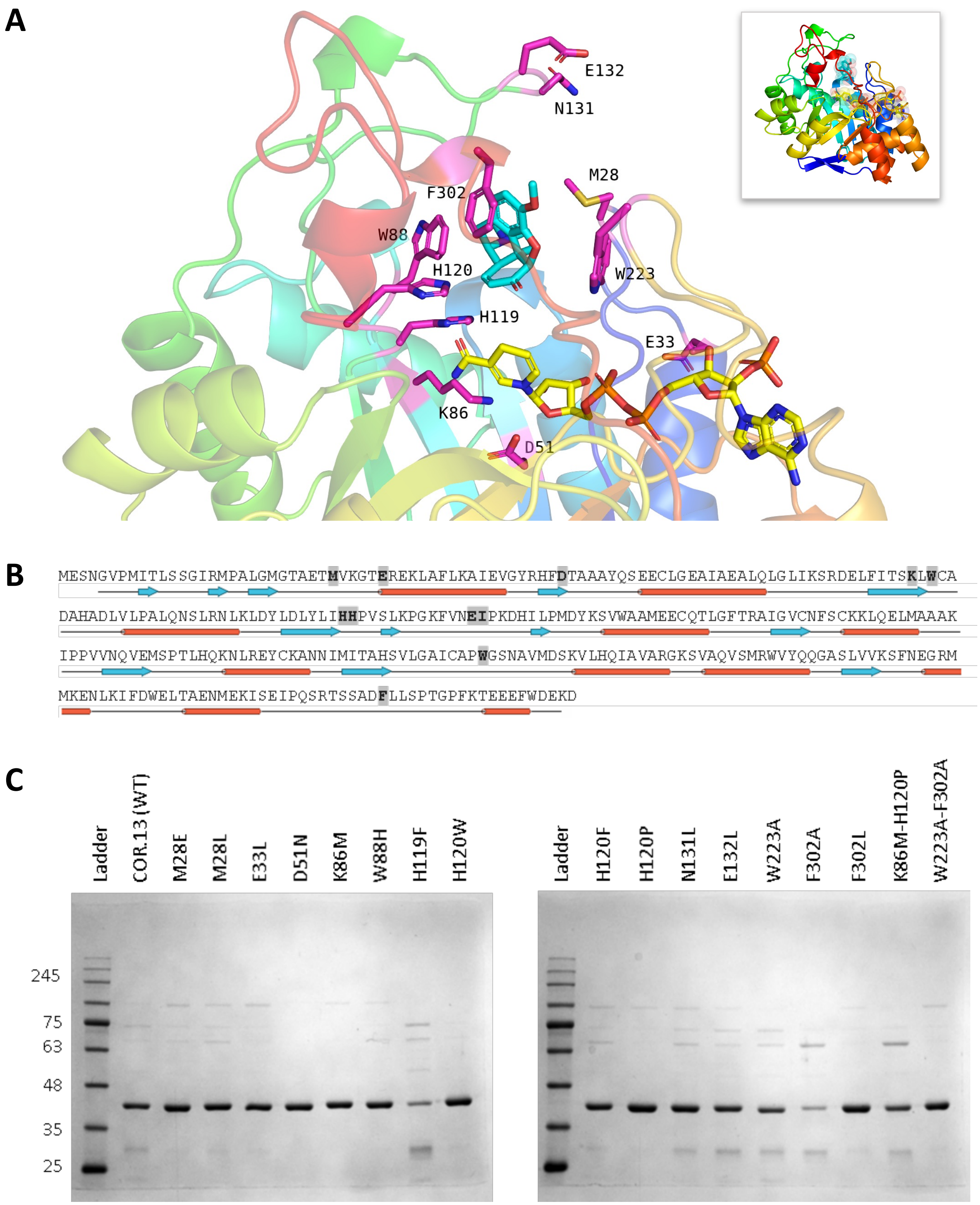

Figure S5 - COR mutagenesis and purification.
A) Approximate three-dimensional position of residues targeted for mutagenesis. Loops with low electron density were modelled using Schrodinger Maestro Prime. Codeinone (cyan) and NADPH (yellow) were docked into the structure using Glide Extra Precision. B) Position of targeted residues (grey) in the COR1.3 polypeptide sequence annotated with empirically-determined secondary structure. C) SDS-PAGE analysis of recombinant COR1.3 protein preparations used for *in vitro* enzyme assays. Gels were stained with Coomassie R-250.

Table S1. Primers used to construct COR mutants. The underlined portion is the region of overlap between forward (F) and reverse (R) primers. Bases in red are those that were mismatched with respect to the native COR sequence in the original plasmid. The three bases in bold are the codon for the residue of interest.

Primer name	Sequence (5' → 3')	T_m (°C)
COR1_3-M28L-F	GAAACGCTGGTCAAAGGCACCGAACGTGAAAAACTG	67
COR1_3-M28L-R	TTTGACCAGCGTTTCAGCCGTGCCCATGCC	70
COR1_3-M28E-F	GAAACGGAAGTCAAAGGCACCGAACGTGAAAAACTG	67
COR1_3-M28E-R	TTTGACTTCCGTTTCAGCCGTGCCCATGCC	70
COR1_3-E33L-F	GGCACCTTACGTGAAAAACTGGCATTTCTGAAAGCTATTG	64
COR1_3-E33L-R	TTCACGTAAGGTGCCTTTGACCATCGTTTCAGCC	63
COR1_3-D51N-F	CATTTCAACACGGCGGCCGCATACCAGAGTGAAG	66
COR1_3-D51N-R	CGCCGTGTTGAAATGGCGATAACCCACTTCAATAGCTTTC	66
COR1_3-K86M-F	ACCTCGATGCTGTGGTGTGCTGATGCACACGC	67
COR1_3-K86M-R	CCACAGCATCGAGGTGATAAACAGTTCGTCACGTGATTTAATCAG	65
COR1_3-W88H-F	AAACTGCATTGTGCTGATGCACACGCAGACCTG	67
COR1_3-W88H-R	AGCACAATGCAGTTTCGAGGTGATAAACAGTTCGTCACG	68
COR1_3-H119F-F	CTGATTTTTCACCCGGTCTCTCTGAAACCGGGC	66
COR1_3-H119F-R	CGGGTGAAAAATCAGGTACAGGTCCAGATAATCCAGTTTC	64
COR1_3-H120F-F	ATTCATTTCCCGGTCTCTCTGAAACCGGGCAAATTCG	68
COR1_3-H120F-R	GACCGGGAAATGAATCAGGTACAGGTCCAGATAATCCAG	66
COR1_3-H120W-F	ATTCATTGGCCGGTCTCTCTGAAACCGGGCAAATTCG	68
COR1_3-H120W-R	GACCGGCCAATGAATCAGGTACAGGTCCAGATAATCCAG	66
COR1_3-H120P-F	ATTCATCCGCCGGTCTCTCTGAAACCGGGCAAATTCG	68
COR1_3-H120P-R	GACCGGCGGATGAATCAGGTACAGGTCCAGATAATCCAG	66
COR1_3-N131L-F	TTCGTGCTGGAAATTCCGAAAGATCATATCCTGCCG	65
COR1_3-N131L-R	AATTTCCAGCACGAATTTGCCCGGTTTCAGAGAG	66
COR1_3-E132L-F	GTGAACCTGATTCCGAAAGATCATATCCTGCCGATGGAC	66
COR1_3-E132L-R	CGGAATCAGGTTCACGAATTTGCCCGGTTTCAGAGAG	66
COR1_3-W223A-F	CCCGGCGGGTAGTAACGCGGTGATGGATTCCAAAG	67
COR1_3-W223A-R	CTACCCGCCGGGGCACAAATCGCGCCCAG	68
COR1_3-F302A-F	TCTGCCGACGCGCTGCTGTCACCGACCGG	69
COR1_3-F302A-R	CGCGTCGGCAGAGCTCGTGCGAGACTGC	67
COR1_3-F302L-F	TCTGCCGACGCGCTGCTGTCACCGACCGG	69
COR1_3-F302L-R	CGCGTCGGCAGAGCTCGTGCGAGACTGC	67
